# Enhancing infection prevention and control in behavioral health settings: barriers, facilitators, and tailored strategies

**DOI:** 10.1017/ash.2025.10290

**Published:** 2026-01-29

**Authors:** Isabelle V. Boullier, Kevin M. Gibas

**Affiliations:** 1 Department of Epidemiology, Brown University School of Public Health, Providence, RI, USA; 2 Department of Medicine, Warren Alpert Medical School of Brown University, Providence, RI, USA; 3 Department of Epidemiology & Infection Prevention, Rhode Island Hospital, Providence, RI, USA

## Abstract

This review examines barriers and facilitators to implementing infection prevention and control (IPC) practices in behavioral health settings. Among 63 studies identified, environmental design, staffing/training limitations, patient behaviors, and therapeutic conflicts were common barriers. Facilitators included targeted training, collaboration, and adaptable IPC policies, underscoring the need for tailored interventions.

## Introduction

Infection prevention and control (IPC) is a fundamental component of patient safety across all healthcare settings, yet successful implementation can vary widely depending on patient population, care environment, and availability of resources. Behavioral health settings pose distinct and often underrecognized IPC challenges arising from the unique physical design of these settings and the inherent nature of psychiatric care.^1,2^ Patients may exhibit behaviors or cognitive conditions that hinder adherence to IPC protocols. Environmental features—such as limited sink access, restricted use of alcohol-based sanitizers, and communal spaces—prioritize safety and therapeutic engagement but complicate effective IPC.^3–5^

Despite extensive research in acute care, evidence and guidance for IPC specific to behavioral healthcare environments is limited. This hinders adoption of feasible, evidence-based practices that balance IPC with therapeutic goals. Continued research is needed to elucidate barriers to implementing and sustaining IPC practices in behavioral health settings and to develop contextually appropriate strategies that promote safe, patient-centered, and sustainable IPC practices. This narrative review synthesizes evidence on barriers and facilitators to IPC in behavioral health settings and identifies opportunities for sustainable, context-specific practices.

## Methods

A literature review was conducted across *PubMed*, *Embase*, and *Web of Knowledge* to identify studies related to IPC in behavioral health settings. Medical Subject Headings and equivalent database-specific terms were used to identify relevant articles (Supplemental Table 1). Inclusion criteria comprised peer-reviewed studies published in English that examined IPC practices, barriers, facilitators, or outbreak management in behavioral health environments. Peer-reviewed empirical studies, review studies, and expert opinion articles were included. Exclusion criteria included studies unrelated to behavioral health or IPC topics, non-peer-reviewed publications, and conference abstracts/presentations (Supplemental Table 2). Eligible articles were screened independently by two reviewers, with discrepancies resolved by consensus. Studies were analyzed for thematic trends related to IPC implementation, adherence, and sustainability.

## Results

**Figure 1.**
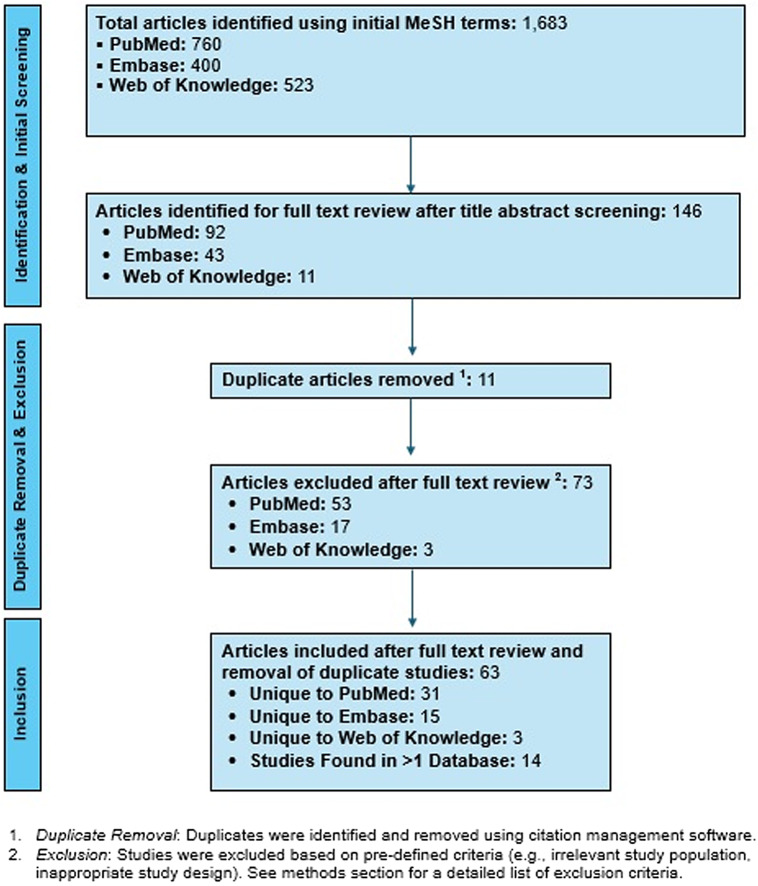
Summary of search and review process.

The initial search identified 1,683 articles, of which 146 underwent full-text review after title and abstract screening (Figure 1). Following full-text assessment, 63 articles met inclusion criteria (Supplemental Figure 3). These studies were analyzed for themes categorized as barriers (environmental, organizational, or behavioral factors impeding IPC implementation/adherence) and facilitators (interventions, resources, or system-level supports promoting IPC implementation/adherence) (Table 1).

**Table 1.** Barriers and facilitators to infection prevention and control (IPC) in behavioral health settings

Barriers		
Themes	Key findings	References % (n)
**Environmental and physical design challenges**	Behavioral health settings are often designed for patient safety and therapeutic engagement rather than infection containment, with communal layouts, shared spaces, and limited capacity for isolation/cohorting. Physical and operational constraints—including lack of negative-pressure or single rooms, private bathrooms, areas for cohorting, and frequent communal activities—hinder implementation of IPC measures and increase the risk of transmission of infectious diseases. Aging infrastructure and limited funding for facility upgrades constrain the ability to implement engineering controls such as ventilation improvements, cohorting areas, or negative-pressure rooms.	81% (n = 51)
**Asymptomatic spread and delayed detection of disease**	Asymptomatic transmission and delayed recognition of illness hinder outbreak containment in behavioral health settings. Patients with psychiatric disorders may have difficulty recognizing or communicating symptoms due to underlying conditions or medication effects, reducing the reliability of clinical screening and delaying identification of cases.	32% (n = 20)
**Behavioral & cognitive challenges of patients**	Patients with severe mental illness or cognitive impairment often had difficulty understanding or adhering to isolation precautions, masking, or physical distancing. Behavioral symptoms such as agitation, impulsivity, or psychosis complicated consistent IPC adherence.	59% (n = 37)
**Staff capacity, compliance, & adherence challenges**	Limited IPC training and familiarity among behavioral health staff compared to other units. Frequent, close staff–patient interactions heighten exposure risk, especially with limited PPE or guidance. Staffing shortages, burnout, and high turnover undermine consistent IPC adherence. Competing clinical demands and misconceptions about infection risk reduce compliance. Restricted sanitizer use, limited sink access, and environmental barriers hinder sustained IPC practice.	60% (n = 38)
**Resource constraints**	Limited PPE, dedicated IPC staff, and IPC training in behavioral health areas may delay outbreak recognition/containment. Resource allocation during outbreaks/emergencies often prioritizes acute medical settings, leaving behavioral health settings with insufficient PPE, testing capacity, and environmental controls.	32% (n = 20)
**Therapeutic and ethical tensions**	IPC measures such as isolation, masking, reduced group therapy, or visitation restrictions often conflict with therapeutic principles of engagement, autonomy, and social interaction central to psychiatric care. Staff face ethical dilemmas balancing patient rights and safety, particularly when enforcing precautions risk escalating agitation or requiring coercive measures.	56% (n = 35)
**Limited integration of IPC in behavioral health**	IPC teams were often peripheral to behavioral health operations, leading to weak coordination, inconsistent policy enforcement, and delayed outbreak response across behavioral health facilities. National and institutional IPC guidelines were often developed for acute medical settings and failed to account for behavioral health–specific limiting effective implementation.	16% (n = 10)
**Need for longitudinal care & monitoring**	Patients with certain psychiatric conditions may require ongoing in-person visits for hospitalization, medication administration (e.g., long-acting injectables), and laboratory monitoring (e.g., clozapine), thus increasing exposure risk. IPC measures that restrict face-to-face contact or routine monitoring can disrupt continuity of psychiatric care, delay treatment, and exacerbate underlying mental health conditions.	16% (n = 10)
**Stigma & fear**	Fear and stigma surrounding infectious diseases can discourage patients, families, and staff from engaging in care, creating barriers to both medical and mental health services.	5% (n = 3)

Facilitators		
Theme	Key findings	References
**Establishing testing, surveillance, & triage protocols**	Structured testing and triage systems—using universal screening and designated quarantine, isolation, and standard-care zones—enabled early case detection and reduced nosocomial transmission across various behavioral health settings. Routine testing of both symptomatic and asymptomatic patients, paired with clear spatial separation, improved outbreak control across various infections (COVID-19, Influenza, Ebola, etc.), while preserving continuity of behavioral health care.	30% (n = 19)
**Establishing protocols & spaces for patient cohorting**	Dedicated spaces for behavioral health patients requiring isolation for transmissible infections provide a practical balance between maintaining essential psychiatric services and strengthening infection prevention and outbreak control efforts for pathogens such as COVID-19 and norovirus.	37% (n = 23)
**Availability of IPC & epidemiology teams**	Dedicated teams involving IPC professionals and behavioral health staff were critical for timely outbreak detection, contact tracing, and identification of transmission pathways in behavioral health settings.	16% (n = 10)
**Enhanced PPE & hand hygiene practices**	Behavioral health–adapted hand hygiene initiatives—such as supervised or directly observed use of alcohol-based hand rubs, staff education, and installation of secure dispensers—were associated with reduced transmission of pathogens including COVID-19, MRSA, and metapneumovirus.	22% (n = 14)
**Behavioral health–adapted IPC policies and integration into psychiatric practice**	Tailoring IPC policies to the behavioral health environment improves adherence and supports a balance between IPC, patient safety, and therapeutic care. Embedding IPC principles into daily psychiatric practice strengthens outbreak preparedness and ensures safe, continuous mental health treatment during infectious disease emergencies.	48% (n = 30)
**Staff training, education, & role modeling**	Targeted, ongoing IPC training—covering PPE use, hand hygiene, and communication strategies—consistently improved staff confidence, compliance, and safety across behavioral health settings. Interdisciplinary education and role modeling by IPC and behavioral health leaders improved adherence, promoted consistent application of evolving IPC policies, and supported continuity of care during outbreaks.	64% (n = 40)
**Interdisciplinary collaboration & coordination**	Close collaboration between behavioral health teams and infection prevention, nursing, facilities, and public health partners was critical for adapting IPC protocols to the unique environment and needs of behavioral health care areas. Facilities with established interdisciplinary preparedness structures—such as those developed during prior outbreaks (e.g., H1N1)—demonstrated more effective and coordinated responses to COVID-19 and subsequent infectious threats.	57% (n = 36)
**Administrative and system-level support**	Strong administrative and system-level support, including leadership engagement, clear communication, and timely resource allocation, was effective in promoting IPC implementation, enabling rapid policy adoption, staff training, and sustained morale in behavioral health settings during outbreaks	11% (n = 7)
**Environmental modifications**	Environmental modifications—such as zoning of clean and contaminated areas, designated entry/exit routes, and structured patient movement—helped reduce cross-contamination while maintaining therapeutic programing. Adjustments to ventilation, cleaning frequency, and space use (e.g., smaller group activities, rearranged communal areas) enhanced infection control without compromising patient safety or ligature precautions. Designing behavioral health units with flexible layouts and capacity for isolation or cohorting was identified as a key strategy to improve future IPC readiness.	32% (n = 20)
**Adapted therapeutic programing & patient engagement**	Modifying group activities to smaller, distanced, or individualized sessions with structured schedules and visual cues to maintain therapeutic engagement while reducing transmission risk. Clear, developmentally tailored communication and behavioral reinforcement promotes patient understanding and adherence to hygiene, IPC protocols, and distancing measures.	13% (n = 8)
**Flexible & innovative service delivery models**	Telepsychiatry, hybrid care, and mobile outreach services reduced exposure risk while maintaining continuity of psychiatric care during outbreaks. Flexible service models, including remote monitoring and community-based crisis alternatives, may help decompress inpatient units and sustain access to behavioral health care.	21% (n = 13)
**Rapid feedback & continuous quality improvement**	Real-time monitoring of infection trends and operational feedback allows facilities to adjust triage, testing, and cohorting processes quickly during outbreaks. Transparent communication with staff and patients promotes trust, improves adherence to IPC measures, and supports therapeutic relationships amid evolving IPC interventions.	5% (n = 3)
**Enhanced communication & transparency**	Regular briefings, clear updates, and ongoing education for staff and patients improve morale, trust, and adherence to IPC practices during rapidly changing conditions. Transparent, culturally sensitive communication with patients, families, and communities reduces stigma and fear, supporting engagement with behavioral health services throughout outbreaks.	21% (n = 13)
**Integration of behavioral health into public health preparedness**	COVID-19 demonstrated the importance of embedding behavioral health within emergency preparedness and IPC planning, ensuring psychiatry’s inclusion in outbreak response and policy development. Increased collaboration between public health and mental health sectors promotes long-term strategies to strengthen patient care, staff resilience, and systemwide preparedness.	13% (n = 8)

Common barriers included environmental and design challenges (81% of articles, n = 51), staff capacity, training, and compliance challenges (60%, n = 38), patient behavioral and cognitive factors (59%, n = 37), and ethical conflicts between IPC measures and therapeutic goals (56%, n = 35). Additional barriers included resource constraints, delayed disease recognition/asymptomatic transmission, limited IPC integration within behavioral health operations, ongoing need for in-person psychiatric monitoring, and stigma/fear surrounding infection.

Frequently cited facilitators included staff training, education, and role modeling (64%, n = 40), interdisciplinary collaboration (57%, n = 36), and behavioral health–adapted IPC policies (48%, n = 30). Other facilitators included structured testing and triage protocols, designated cohorting spaces and protocols, enhanced PPE and hygiene programs/policies, environmental modifications, adoption of flexible care models, real-time feedback, leadership engagement, established IPC teams, enhanced communication/transparency, and integration of behavioral health into public health planning.

## Discussion

This review highlights that IPC in behavioral health settings remains an underdeveloped and understudied area. Across the published literature, a consistent theme is that conventional IPC frameworks—largely derived from acute care contexts—do not adequately reflect the structural, behavioral, therapeutic, and ethical complexities inherent to behavioral healthcare environments. Notably, much of the existing research regarding IPC in behavioral health environments was generated in response to the COVID-19 pandemic, which not only illuminated long-standing systemic vulnerabilities but also encouraged innovation, cross-disciplinary collaboration, and renewed attention to IPC practices in this historically understudied area.

Multiple publications underscore that behavioral healthcare settings face unique environmental and operational barriers to IPC. These environments are intentionally constructed for safety, visibility, and communal engagement rather than infection containment. Communal therapeutic areas, shared living spaces and bathrooms, and limited capacity for isolation make conventional IPC interventions—such as cohorting, distancing, and negative pressure ventilation—difficult to implement and sustain and increases the risk of spread of communicable diseases.^1,2,6–8^ During outbreaks of infections with respiratory and enteric pathogens, these features were often linked to delayed containment and ongoing transmission. These structural and operational factors often created tension between IPC priorities and the ethical tenets of psychiatric care, where patient autonomy and maintenance of therapeutic rapport are essential.

Patient-related factors, such as, underlying medical conditions, medications, and underlying mental illness, compound these challenges, compound these challenges. Individuals with severe mental illness, dementia, or cognitive impairment may have limited insight or difficulty adhering to masking, hygiene, or isolation protocols and have challenges with symptom recognition/reporting. Behavioral symptoms such as agitation, impulsivity, or psychosis can necessitate close contact or physical restraint, elevating transmission risk for staff and other patients.^1,3,7^ Compounding these risks, staff often report barriers related to training, resources, and institutional support resulting in suboptimal staff compliance with IPC recommendations.^1,9^ Several studies reported that staff often lacked adequate IPC training, had limited access to PPE, and received unclear guidance on IPC protocols.^3,4,8^ Staffing shortages, burnout, and restricted availability of alcohol-based hand rubs further undermined IPC sustainability.^2,4,10^

Despite extensive IPC challenges inherent to behavioral health settings, numerous studies found that adaptive, interdisciplinary strategies can effectively facilitate implementation and adherence to IPC best practices. Interdisciplinary collaboration, particularly between infection prevention, psychiatry, nursing, and facilities teams, consistently emerged as a critical facilitator of IPC success in these settings.^4^ Programs that embedded IPC specialists within behavioral health governance structures achieved more coordinated outbreak responses, stronger policy enforcement, and improved cross-disciplinary communication.^4^ Complementary strategies—such as ongoing staff education, leadership engagement, and behavioral health–adapted IPC policies—further strengthened compliance.^1,3,9^ Transparent communication with staff and patients, coupled with the integration of IPC principles into regular psychiatric operations, also supported sustained adherence.^1,8^

During infectious outbreaks, several adaptive strategies proved effective in supporting IPC implementation. Structured testing protocols, universal admission screening, and flexible spatial arrangements enabled facilities to balance IPC priorities with therapeutic needs.^5,6,10^ Cohorting and “three-space” triage models—separating new admissions, confirmed cases, and noninfected units—were associated with decreased nosocomial transmission.^6,8,10^ Environmental modifications, including enhanced ventilation, spatial zoning, and digital surveillance systems, demonstrated feasibility without compromising safety or therapeutic goals.^5,7,8^ Data published during the COVID-19 pandemic found that technology-enabled interventions—including telepsychiatry, hybrid service delivery, and real-time feedback systems—supported care continuity while minimizing exposure risk.

This review has several limitations. The included articles were heterogeneous in design and quality, limiting generalizability. Publication bias likely favored outbreak and intervention reports over routine IPC practices, and most studies originated from high-income countries, leaving gaps in data from community and low-resource settings. No formal risk-of-bias assessment was conducted. Despite these, this review provides a comprehensive synthesis of themes and evidence gaps related to IPC in behavioral health environments.

This review highlights the scarcity of data regarding IPC best practices in behavioral health settings. Most evidence remains descriptive, underscoring the need for further research to inform behavioral health-specific IPC models. Effective IPC in behavioral health depends on tailoring approaches to the unique clinical, environmental, and psychosocial context of these settings. Future priorities should include developing evidence-based IPC guidelines for behavioral health settings, integrating behavioral health into IPC governance structures, and investing in environmental modifications and infrastructure that supports both safety and therapeutic goals.

## Supporting information

10.1017/ash.2025.10290.sm001 Boullier and Gibas supplementary material 1 Boullier and Gibas supplementary material

10.1017/ash.2025.10290.sm002 Boullier and Gibas supplementary material 2 Boullier and Gibas supplementary material

10.1017/ash.2025.10290.sm003 Boullier and Gibas supplementary material 3 Boullier and Gibas supplementary material

## Supplementary material

The supplementary material for this article can be found at https://doi.org/10.1017/ash.2025.10290.

## Previous presentation of the data/findings

None/Not applicable.
