# Supplementary material for: Enhancing infection prevention and control in behavioral health settings: barriers, facilitators, and tailored strategies
Source: Antimicrob Steward Healthc Epidemiol. 2026 Jan 29;6(1):e36. doi: 10.1017/ash.2025.10290 (PMC12854875; doi:10.1017/ash.2025.10290)
Supplement: Boullier and Gibas supplementary material 1 — Boullier and Gibas supplementary material [file S2732494X25102908sup001.docx]

| **Supplemental Figure 2. Inclusion & Exclusion Criteria** | |
| --- | --- |
| **Inclusion Criteria** | - **Population/Setting:** Studies conducted in behavioral health care environments—such as psychiatric hospitals, inpatient psychiatric units within general hospitals, mental health facilities, or other specialized behavioral health settings—or studies involving patient populations receiving behavioral health or psychiatric care. - **Topic/Content:** Articles/studies that examine infection prevention and control (IPC) practices, barriers, facilitators, or outbreak management in behavioral health or psychiatric populations or care settings. - **Study Type:** Peer-reviewed empirical research studies (observational, interventional, cross-sectional, case reports/series, outbreak investigations, qualitative studies, or mixed-methods) and relevant systematic/narrative reviews and peer-reviewed letters by key opinion leaders. - **Language:** Published in English. - **Timeframe:** No date restrictions. |
| **Exclusion Criteria** | - **Population/Setting:** Studies/articles without specific relevance to psychiatric or behavioral health settings or populations. Studies/articles not addressing infection prevention, control, outbreak management, or related safety protocols (e.g., those focused only on psychiatric treatment, medication management, patient mental health, or non-IPC safety measures). - **Publication Type:** Non-peer reviewed publications, abstracts without full data, lectures/presentations, and unpublished dissertations/theses. - **Language**: Non-English articles. - **Articles/Studies with Insufficient Detail:** Articles that mention IPC superficially without describing barriers, facilitators, or interventions in a behavioral health context. |
