# Supplementary material for: Enhancing infection prevention and control in behavioral health settings: barriers, facilitators, and tailored strategies
Source: Antimicrob Steward Healthc Epidemiol. 2026 Jan 29;6(1):e36. doi: 10.1017/ash.2025.10290 (PMC12854875; doi:10.1017/ash.2025.10290)
Supplement: Boullier and Gibas supplementary material 2 — Boullier and Gibas supplementary material [file S2732494X25102908sup002.docx]

**Supplemental Figure 3: List of articles identified from the literature review search**

1. Akbari K, Zareiyan A, Yari A, Najafi M, Azizi M, Ostadtaghizadeh A. Mental health preparedness and response to epidemics focusing on COVID-19 pandemic: a qualitative study in Iran. BMC Public Health. 2024;24(1):1980. Published 2024 Jul 24. doi:10.1186/s12889-024-19526-2
2. Angelino AF, Lyketsos CG, Ahmed MS, Potash JB, Cullen BA. Design and Implementation of a Regional Inpatient Psychiatry Unit for Patients who are Positive for Asymptomatic SARS-CoV-2. Psychosomatics. 2020;61(6):662-671. doi:10.1016/j.psym.2020.06.018
3. Aziz AM. COVID-19 outbreak management in a mental healthcare setting. Br J Nurs. 2022;31(9):470-476. doi:10.12968/bjon.2022.31.9.470
4. Balfour ME, Curtis A, Miller AH. Ensuring Access to Crisis Services During COVID-19. Psychiatr Serv. 2020;71(11):1208. doi:10.1176/appi.ps.72303
5. Barnett B, Esper F, Foster CB. Keeping the wolf at bay: Infection prevention and control measures for inpatient psychiatric facilities in the time of COVID-19. Gen Hosp Psychiatry. 2020 Sep-Oct;66:51-53. doi: 10.1016/j.genhosppsych.2020.07.004. Epub 2020 Jul 12. PMID: 32682153; PMCID: PMC7354767.
6. Bhome R, Huntley J, Dalton-Locke C, et al. Impact of the COVID-19 pandemic on older adults mental health services: A mixed methods study. Int J Geriatr Psychiatry. 2021;36(11):1748-1758. doi:10.1002/gps.5596
7. Brody BD, Shi Z, Shaffer C, et al. Universal COVID-19 testing and a three-space triage protocol is associated with a nine-fold decrease in possible nosocomial infections in an inpatient psychiatric facility. Psychiatry Res. 2021;302:114036. doi:10.1016/j.psychres.2021.114036
8. Bwire G, Malimbo M, Kagirita A, et al. Nosocomial Cholera Outbreak in a Mental Hospital: Challenges and Lessons Learnt from Butabika National Referral Mental Hospital, Uganda. Am J Trop Med Hyg. 2015;93(3):534-538. doi:10.4269/ajtmh.14-0730
9. Cheng, V.C.C., A.K.L. Wu, C.H.Y. Cheung, et al. Outbreak of human metapneumovirus infection in psychiatric inpatients: implications for directly observed use of alcohol hand rub in prevention of nosocomial outbreaks. Journal of Hospital Infection. 2007;67: 336–343. doi:10.1016/j.jhin.2007.09.010.
10. Chun JY, Jun JY, Choi J, et al. Coronavirus Disease 2019 Outbreak in a Psychiatric Closed Ward: What We Have to Learn. Front Psychiatry. 2021;11:579235. Published 2021 Jan 22. doi:10.3389/fpsyt.2020.579235
11. Constantino-Shor C, Rani G, Olin S, Holmes C, Nasenbeny K. Containment of a COVID-19 Outbreak in an Inpatient Geriatric Psychiatry Unit. J Am Psychiatr Nurses Assoc. 2021;27(1):77-82. doi:10.1177/1078390320970653
12. Das A, Sawyer AT, Bailey AK, Green JF, Allen L. A Virtual Inpatient Psychiatry Unit in a Multicampus Health Care System During the COVID-19 Pandemic. Psychiatr Serv. 2023;74(6):663-666. doi:10.1176/appi.ps.20220299
13. Duden GS, Gersdorf S, Stengler K. Global impact of the COVID-19 pandemic on mental health services: A systematic review. J Psychiatr Res. 2022;154:354-377. doi:10.1016/j.jpsychires.2022.08.013
14. Ebner W, Schlachetzki J, Schneider C, Dettenkofer M, Langosch JM. Hand hygiene seems to be sufficient for prevention of MRSA transmission on a closed psychiatric ward. J Hosp Infect. 2010;75(4):334-335. doi:10.1016/j.jhin.2010.02.010
15. Falgione J, Snyder GM, Cannon C, Huss JM. Assessing COVID-19 transmission risk: Roommate and unit mate exposures at an inpatient behavioral health facility. Am J Infect Control. 2025;53(1):110-114. doi:10.1016/j.ajic.2024.09.003
16. Fearon P. Opening up while locking down: how an Irish independent sector mental health service is responding to the COVID-19 crisis. Ir J Psychol Med. 2020;37(3):172-177. doi:10.1017/ipm.2020.68
17. Fukuta Y, Muder RR. Infections in psychiatric facilities, with an emphasis on outbreaks. Infect Control Hosp Epidemiol. 2013;34(1):80-88. doi:10.1086/668774
18. Galloway A, Roberts C, Hunt EJ. An outbreak of Salmonella typhimurium gastroenteritis in a psychiatric hospital. J Hosp Infect. 1987;10(3):248-254. doi:10.1016/0195-6701(87)90005-3
19. Gilbride SJ, Lee BE, Taylor GD, Forgie SE. Successful containment of a norovirus outreak in an acute adult psychiatric area. Infect Control Hosp Epidemiol. 2009;30(3):289-291. doi:10.1086/595733
20. Hatch RJ. Descriptive study of nosocomial infections in a short-term inpatient behavioral health setting. Am J Infect Control. 2002 Feb;30(1):67-8. doi: 10.1067/mic.2002.121553. PMID: 11852421
21. Herman A, Filip K, Wanke-Rytt M, Wolańczyk T. Practical guidelines for management of patients with suspected or confirmed COVID-19 hospitalized in a child and adolescent psychiatric ward. Psychiatr Pol. 2022 Feb 27;56(1):115-122. English, Polish. doi: 10.12740/PP/OnlineFirst/124899. Epub 2022 Feb 27. PMID: 35569152.
22. Hernández-Huerta D, Alonso-Sánchez EB, Carrajo-Garcia CA, Montes-Rodríguez JM. The impact of COVID-19 on acute psychiatric inpatient unit. Psychiatry Res. 2020;290:113107. doi:10.1016/j.psychres.2020.113107
23. Houben F, van Hensbergen M, den Heijer CDJ, Dukers-Muijrers NHTM, Hoebe CJPA. Barriers and facilitators to infection prevention and control in Dutch psychiatric institutions: a theory-informed qualitative study. BMC Infect Dis. 2022;22(1):243. Published 2022 Mar 11. doi:10.1186/s12879-022-07236-2
24. Hsu ST, Chou LS, Chou FH, et al. Challenge and strategies of infection control in psychiatric hospitals during biological disasters-From SARS to COVID-19 in Taiwan. Asian J Psychiatr. 2020;54:102270. doi:10.1016/j.ajp.2020.102270
25. Huang FL, Chen PY, Shi ZY, Chan CH, Huang SK. An unusual respiratory syncytial virus nosocomial outbreak in an adult psychiatry ward. Jpn J Infect Dis. 2009;62(1):61-62.
26. Huang MH, Hung CY, Su TP. Prepare the psychiatric inpatient unit for COVID-19 pandemic. J Chin Med Assoc. 2022;85(7):739-740. doi:10.1097/JCMA.0000000000000753
27. Hubiche T, Brazier C, Vabret A, Reynaud S, Roudiere L, Del Giudice P. Measles Transmission in a Fully Vaccinated Closed Cohort: Data From a Nosocomial Clustered Cases in a Teenage Psychiatric Unit. Pediatr Infect Dis J. 2019;38(9):e230-e232. doi:10.1097/INF.0000000000002372
28. Hughes FA. H1N1 pandemic planning in a mental health residential facility. J Psychosoc Nurs Ment Health Serv. 2010;48(3):37-41. doi:10.3928/02793695-20100202-02
29. Lau LHW, Lam QSK, Siu MMY, Tang TSK, Suen LKP, Lam SC. Compliance of healthcare workers in a psychiatric inpatient ward to infection control practices during the COVID-19 pandemic: a participant observation study supplemented with a self-reported survey. BMC Infect Dis. 2024;24(1):592. Published 2024 Jun 17. doi:10.1186/s12879-024-09429-3
30. Levitt GA. Infection control for MRSA in a psychiatric hospital. Gen Hosp Psychiatry. 2014;36(4):422-424. doi:10.1016/j.genhosppsych.2014.02.005
31. Li L. Challenges and Priorities in Responding to COVID-19 in Inpatient Psychiatry. Psychiatr Serv. 2020;71(6):624-626. doi:10.1176/appi.ps.202000166
32. Li PH, Wang SY, Tan JY, Lee LH, Yang CI. Infection preventionists' challenges in psychiatric clinical settings. Am J Infect Control. 2019;47(2):123-127. doi:10.1016/j.ajic.2018.08.010
33. Lien M.-H., Kuo H.-Y., Liang H.-C., Huang M.-L., Liu Y.-C., Huang P.-T. To reduce the incidence of upper respiratory tract infection in psychiatric patients in a regional teaching hospital experience
34. Liu KY, Kulatilake A, Kalafatis C, et al. Infection control and the prevalence, management and outcomes of SARS-CoV-2 infections in mental health wards in London, UK: lessons learned from wave 1 to wave 2. BJPsych Open. 2022;8(2):e63. Published 2022 Mar 8. doi:10.1192/bjo.2022.31
35. London S. COVID-19, Autonomy, and the Inpatient Psychiatric Unit. Acad Psychiatry. 2020;44(6):671-672. doi:10.1007/s40596-020-01314-w
36. Ma J, Zhong H, Jiang M, et al. Emergency response strategy for containing COVID-19 within a psychiatric specialty hospital in the epicenter of the COVID-19 epidemic in China. Transl Psychiatry. 2020;10(1):268. Published 2020 Aug 4. doi:10.1038/s41398-020-00959-3
37. McGloin JM, Asokaraj N, Feeser B, et al. Coronavirus disease 2019 (COVID-19) outbreak on an inpatient psychiatry unit: Mitigation and prevention. Infect Control Hosp Epidemiol. 2022;43(9):1290-1291. doi:10.1017/ice.2021.233
38. Mohammed A, Sheikh TL, Poggensee G, et al. Mental health in emergency response: lessons from Ebola. Lancet Psychiatry. 2015;2(11):955-957. doi:10.1016/S2215-0366(15)00451-4
39. Murdoch S. A safe environment for care: infection control nurses' role in mental health units. Mental Health Practice. 2004;8(2):22-24
40. Nichols J, Gannon JM, Conlogue J, et al. Ensuring care for clozapine-treated schizophrenia patients during the COVID-19 pandemic. Schizophr Res. 2020;222:499-500. doi:10.1016/j.schres.2020.05.053
41. Nováková V, Cantero-Caballero M, Zoni AC, Plá-Mestre R, Olmedo-Lucerón Mdel C, Rodríguez-Pérez P. Epidemic keratoconjunctivitis outbreak in a closed psychiatric ward. Infect Control Hosp Epidemiol. 2013;34(7):764-765. doi:10.1086/671007
42. Nystazaki M, Alevizopoulos G. Clozapine treatment: Ensuring ongoing monitoring during the COVID-19 pandemic. Psychiatriki. 2021;32(2):165-166. doi:10.22365/jpsych.2021.005
43. Paletta A, Yu D, Li D, Sareen J. COVID-19 pandemic inpatient bed allocation planning - A Canada-wide approach. Gen Hosp Psychiatry. 2021;69:126-128. doi:10.1016/j.genhosppsych.2020.12.015
44. Parrish E. The next pandemic: COVID-19 mental health pandemic. Perspect Psychiatr Care. 2020 Jul;56(3):485. doi: 10.1111/ppc.12571. PMID: 32602165.
45. Patel S, Gautam M, and Mahr G. COVID-19 and Infection Control: A Perspective From the Psychiatric Ward. Prim Care Companion CNS Disord 2020; 22(3).
46. Pinals DA, Hepburn B, Parks J, Stephenson AH. The Behavioral Health System and Its Response to COVID-19: A Snapshot Perspective. Psychiatr Serv. 2020;71(10):1070-1074. doi:10.1176/appi.ps.202000264
47. Quidley-Rodriguez N, de Tantillo L. Preventing COVID-19 Infection in Mental Health Units: Recommendations for Best Practices. Issues Ment Health Nurs. 2020;41(11):969-975. doi:10.1080/01612840.2020.1820646
48. Rovers JJE, van de Linde LS, Kenters N, et al. Why psychiatry is different - challenges and difficulties in managing a nosocomial outbreak of coronavirus disease (COVID-19) in hospital care. Antimicrob Resist Infect Control. 2020;9(1):190. Published 2020 Dec 1. doi:10.1186/s13756-020-00853-z
49. Sarcevic N, Popiel M. Maintaining a Bronx inpatient psychiatry service at full capacity during the COVID-19 pandemic. Perspect Psychiatr Care. 2021;57(4):2024-2029. doi:10.1111/ppc.12751
50. Schultz KM, Miller PB, Stancill L, et al. Strategies utilized to prevent and control SARS-CoV-2 transmission in two congregate, psychiatric healthcare settings during the pandemic. Am J Infect Control. 2022;50(5):536-541. doi:10.1016/j.ajic.2022.02.013
51. Shalev D, Shapiro PA. Epidemic psychiatry: The opportunities and challenges of COVID-19. Gen Hosp Psychiatry. 2020 May-Jun;64:68-71. doi:10.1016/j.genhosppsych.2020.03.009. Epub 2020 Apr 3. PMID: 32279023; PMCID:PMC7194518.
52. Sharma A, Sasser T, Schoenfelder Gonzalez E, Vander Stoep A, Myers K. Implementation of Home-Based Telemental Health in a Large Child Psychiatry Department During the COVID-19 Crisis. J Child Adolesc Psychopharmacol. 2020 Sep;30(7):404-413. doi: 10.1089/cap.2020.0062. Epub 2020 Jul 8. PMID: 32639849.
53. Smith TE, Erlich M, Casoy F, Berezin J: The impact of COVID-19 on psychiatric services and individuals with serious mental illness in New York State during 2020. The Journal of Mental Health Policy and Economics 2022; 25(Suppl 1):S30
54. Spitzer Sverd S, Gardner LE, Cabassa JA, et al. A Bronx tale: Exposure, containment and care on inpatient psychiatry units during COVID-19. Gen Hosp Psychiatry. 2021;69:121-123. doi:10.1016/j.genhosppsych.2020.07.010
55. Thompson AD, Berkman ER, Simmons SW, et al. Ethical Considerations in Balancing Use of Seclusion and Restraint With Risk of COVID-19 Exposure: Recommendations for Youth Inpatient Psychiatry Units. J Am Acad Child Adolesc Psychiatry. 2022;61(11):1319-1321. doi:10.1016/j.jaac.2022.04.016
56. Thompson JW Jr, Mikolajewski AJ, Kissinger P, et al. An Epidemiologic Study of COVID-19 Patients in a State Psychiatric Hospital: High Penetrance With Early CDC Guidelines. Psychiatr Serv. 2020;71(12):1285-1287. doi:10.1176/appi.ps.202000270
57. Waitz C, Kaufman K, Caracansi A, Campbell EJ, Ibeziako P. Operational Changes on Child and Adolescent Acute Psychiatric Treatment Programs During COVID-19. J Am Acad Child Adolesc Psychiatry. 2023;62(6):611-613. doi:10.1016/j.jaac.2023.02.007
58. Weber DJ, Sickbert-Bennett EE, Vinjé J, et al. Lessons learned from a norovirus outbreak in a locked pediatric inpatient psychiatric unit. Infect Control Hosp Epidemiol. 2005;26(10):841-843. doi:10.1086/502504
59. Wise ME, Marquez P, Sharapov U, et al. Outbreak of acute hepatitis B virus infections associated with podiatric care at a psychiatric long-term care facility. Am J Infect Control. 2012;40(1):16-21. doi:10.1016/j.ajic.2011.04.331
60. Yang M, Zhu X, Yan F, et al. Digital-based emergency prevention and control system: enhancing infection control in psychiatric hospitals. BMC Med Inform Decis Mak. 2025;25(1):7. Published 2025 Jan 6. doi:10.1186/s12911-024-02809-4
61. Zhang N, Wu K, Wang W. Timely mental health services contribute to the containment of COVID-19 pandemic in China. Glob Health Res Policy. 2020 Sep 3;5:40. doi: 10.1186/s41256-020-00168-x. PMID: 32905275; PMCID: PMC7467755.
62. Zhao W, Jian W, Li H. Preventing and Controlling Measures of 2019 Coronavirus Disease (COVID-19): Practice in Psychogeriatric Ward. Am J Geriatr Psychiatry. 2020;28(7):786-787. doi:10.1016/j.jagp.2020.04.020
63. Zimmerman M, Terrill D, D'Avanzato C, Tirpak JW. Telehealth Treatment of Patients in an Intensive Acute Care Psychiatric Setting During the COVID-19 Pandemic: Comparative Safety and Effectiveness to In-Person Treatment. J Clin Psychiatry. 2021 Mar 16;82(2):20m13815. doi: 10.4088/JCP.20m13815. PMID: 33989463.
