# Supplementary material for: Enhancing infection prevention and control in behavioral health settings: barriers, facilitators, and tailored strategies
Source: Antimicrob Steward Healthc Epidemiol. 2026 Jan 29;6(1):e36. doi: 10.1017/ash.2025.10290 (PMC12854875; doi:10.1017/ash.2025.10290)
Supplement: Boullier and Gibas supplementary material 3 — Boullier and Gibas supplementary material [file S2732494X25102908sup003.docx]

**Supplemental Table 1**. MeSH and Search Terms for Database Search

| **Database** | **Search Terms** |
| --- | --- |
| PubMed | ("Infection Control"[MeSH Terms] OR  "Infection Control/methods"[MeSH Terms] OR  "Cross Infection/prevention and control"[MeSH Terms] OR  "Hospital Acquired Infections/prevention and control"[MeSH Terms] OR "Disease Outbreaks/prevention and control"[MeSH Terms])  **AND**  ("Psychiatric Department, Hospital"[MeSH Terms] OR  "Mental Health Services"[MeSH Terms] OR  "Psychiatric Hospitals"[MeSH Terms] OR  "Inpatients"[MeSH Terms]) |
| Embase | 'infection control'/de OR 'prevention and control'/de OR 'cross infection'/de OR 'healthcare associated infection'/de  **AND**  'psychiatric department'/de OR 'mental health service'/de |
| Web of Knowledge | TS=(Infection Control) OR TS=(Infection Control/methods) OR TS=(Cross Infection/prevention and control) OR  TS=(Hospital Acquired Infections/prevention and control) OR TS=(Disease Outbreaks/prevention and control)  **AND**  TS=(psychiatric department) OR TS=(mental health services) |
